# Enzymatic Processes in Marine Biotechnology

**DOI:** 10.3390/md15040093

**Published:** 2017-03-25

**Authors:** Antonio Trincone

**Affiliations:** Istituto di Chimica Biomolecolare, Consiglio Nazionale delle Ricerche, Via Campi Flegrei, 34, 80078 Pozzuoli, Naples, Italy; antonio.trincone@icb.cnr.it; Tel.: +39-081-867-5095

**Keywords:** marine enzymes, biocatalysts, bioprocesses, biorefinery, seafood, marine biomarkers

## Abstract

In previous review articles the attention of the biocatalytically oriented scientific community towards the marine environment as a source of biocatalysts focused on the habitat-related properties of marine enzymes. Updates have already appeared in the literature, including marine examples of oxidoreductases, hydrolases, transferases, isomerases, ligases, and lyases ready for food and pharmaceutical applications. Here a new approach for searching the literature and presenting a more refined analysis is adopted with respect to previous surveys, centering the attention on the enzymatic process rather than on a single novel activity. Fields of applications are easily individuated: (i) the biorefinery value-chain, where the provision of biomass is one of the most important aspects, with aquaculture as the prominent sector; (ii) the food industry, where the interest in the marine domain is similarly developed to deal with the enzymatic procedures adopted in food manipulation; (iii) the selective and easy extraction/modification of structurally complex marine molecules, where enzymatic treatments are a recognized tool to improve efficiency and selectivity; and (iv) marine biomarkers and derived applications (bioremediation) in pollution monitoring are also included in that these studies could be of high significance for the appreciation of marine bioprocesses.

## 1. Introduction

Currently there is enormous interest in marine biotechnology with the worldwide flourishing of editorial initiatives (journals, books, etc.) hosting important experimental results and surveys from several projects, especially those belonging to the FP7 program and to the topic “Blue growth” of H2020, potentially providing clues that will aid development of enabling technologies in the field. The oceans are the world’s largest ecosystem, covering more than 70% of the earth’s surface. They host the greatest diversity of life in unexplored habitats. The Census of Marine Life, evaluating marine biodiversity, ascertained that at least 50% and potentially more than 90% of marine species are undescribed by science [1]. Only a deep understanding of the complexity of the marine ecosystem will enable human beings to protect the oceans and organisms populating them, and pave the way for sustainable exploitation of marine resources. This knowledge will certainly fuel various applications and itself constitutes the core of marine biotechnology. Increasingly labeled “blue biotechnology”, this wide field covers many aspects that are highly relevant to societal challenges, as is well established in the EU Framework Programme Horizon 2020. Several outlined emerging technologies are (i) robotics; (ii) miniaturized solutions for marine monitoring; (iii) biomimetics; (iv) acoustics; (v) nanobiotechnlogy; (vi) renewable energy harvesting (wave energy, algae biofuels); and (vii) high-performance computing. However, many challenges remain, including a deep comprehension of the “marine biotechnology landscape” and a multidisciplinary approach, not only in education and training [2].

Marine sources (microorganisms in general and symbionts in particular, extremophiles, fungi, plants and animals) are of scientific interest in that the origin of marine biomolecules (i.e., biocatalysts) features all marine bioprocesses. Knowledge of these biocatalysts and their habitat-related properties such as salt tolerance, hyperthermostability, barophilicity and cold adaptivity (of great interest for industry) is necessary for bioprocesses exploitation. One of the most explicative aspects is related to the stereo-chemical properties of a marine enzyme. Substrate specificity and affinity, as evolved properties that are linked to the metabolic functions of the enzymes, are key aspects. Two review articles already appeared in 2010 [3] and in 2011 [4] with different focuses on these topics. The first [3], to draw the attention of the biocatalytically oriented scientific community to the marine environment as a source of biocatalysts; in fact the discussion was mainly about the specific diversity of molecular assets of biocatalysis that were recognized with respect to terrestrial counterparts. The second review [4] spotlighted habitat-related properties from a biochemical point of view, also reporting on important examples in bioprocesses. Various updates of these former analyses of the literature have also recently been published, such as the one by Lima et al. 2016 [5] including marine examples of oxidoreductases, hydrolases, transferases, isomerases, ligases and lyases ready for food and pharmaceutical applications.

In the present review, a new approach for searching the literature and presenting a more refined analysis is adopted with respect to previous surveys. The focus of the literature search is centered on the enzymatic process more than on a single novel activity. This survey is developed according to the biotechnological field of applications where bioprocesses, based on marine enzymes and/or marine biomasses, are central. Focusing on enzymatic processes rather than on single activities helsd us to recognize the fields of application. For the first, a biorefinery value-chain, the provision of biomass is one of the most important aspects, with aquaculture as the prominent sector. In the food industry the interest in the marine domain is similarly developed to deal with the enzymatic procedures adopted in food manipulation. Moreover, as for the selective and easy extraction/modification of structurally complex marine molecules, enzymatic treatments are a recognized tool to improve efficiency and selectivity in fine chemistry processes to get access to bioactive compounds and provide complex core blocks ready for hemisynthesis. In closing, the field of marine biomarkers and derived applications (bioremediation) in pollution monitoring could be of high significance for the appreciation of marine bioprocesses. 

In the fields indicated above, the selected primary articles are presented in tabulated form, picking up different aspects of importance in short explicative notes to avoid a huge amount of text. Selected modern review articles are listed under each paragraph to depict the present state of the art of the related field.

## 2. Literature Search

A survey of the literature has been conducted mainly by using the database ScienceDirect with access to 3800 scientific journals in major scientific disciplines. The search was based on two terms: (i) “enzymatic processing” (in abstract, title or keywords) and (ii) “marine” (in all fields). How to manipulate these queries to get an effective result in terms of the number of hits was first investigated using the search functions offered by the database. In particular, the W/in function (proximity operator) has been found useful for the first query, with the keywords used in the query sorted into phrases (low numbers), sentences (medium) and paragraphs (high) of the hit. This result is then refined by using the AND operator with the word “marine” in all fields. The resulting pattern of these searches is as follows: W/3 (137 hits), W/4 (153), W/5 (161), W/15 (256) and for W/50 (442). As a comparison, a more general coverage alternative search was also used, adopting the same keywords, in the Scopus database. It resulted in a similar score (478 hits), thus confirming the choice of ScienceDirect as the reference database when using W/50. This has been the value adopted for searching for articles for this review. 

**Figure 1:**
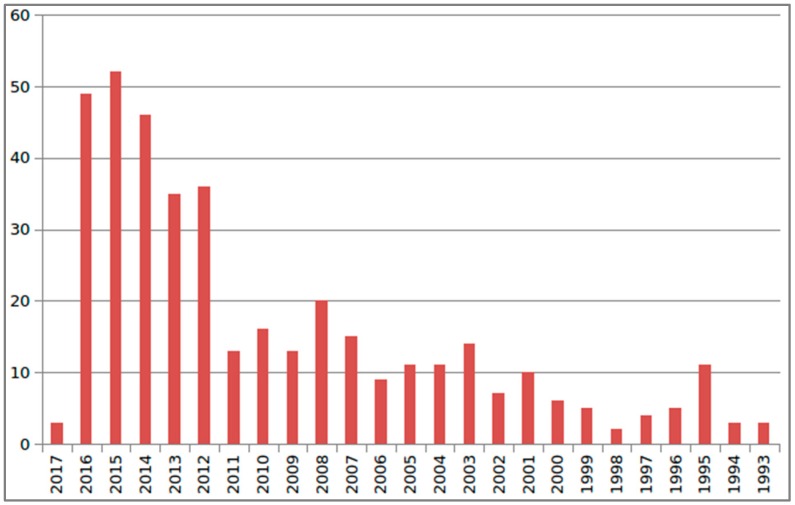
Yearly distribution of the number of hits in the search for articles in this review (see text for details). Fewer than 20 hits per year characterizes the interval 1993–2011; a doubled value is seen for 2012–2016.

An interesting detail is the yearly distribution of the hits (shown in Figure 1 below). Two time intervals can be easily recognized; published results are doubled yearly from 2012 to 2016 with respect to the previous score in the interval 1993 to 2011, characterized by fewer than 20 hits per year. This picture reflects the strategic efforts of various funded programs created by the European Commission to support and foster research in the European Research Area and similar actions in other parts of the world. That logical reason for the variation of the results in the two intervals 1993–2011 and 2012–2016 further confirms the suitability of the keywords adopted.

## 3. Biorefinery

A future sustainable economy based on renewable resources is the main point of the concept of a biorefinery. Research and development studies have been underway for many years in different parts of the world to replace a large fraction of fossil resources. The most important aspect of a biorefinery value-chain is the provision of biomass with a consistent and regular supply of renewable carbon-based raw materials [6]. One of the sectors providing these feedstocks is aquaculture (algae and seaweeds), which, together with biochemical processes (marine enzymes in pre-treatment) adopted, is an important aspect of the domain of marine biotechnology. Needless to say, the focus in many review articles [7] is on the importance of extremophiles and thermostable enzymes to overcome the limitations of biocatalysts in current bioprocesses for lignocellulosic biomass conversion. In this context, it is also of interest to mention the features of marine biocatalysts related to the ecological features of the habitat in which marine organisms thrive. Generally the resulting enzymatic properties are very important from a biotechnological point of view [4,8].

The idea here is to depict both the state of the art about marine enzyme-based bioprocesses and the importance of marine-originating feedstocks in biorefinery. Therefore, biocatalysts and biomass are the two fundamental elements on which the analysis of primary articles in the literature is based here (Table 1 [9,10,11,12,13,14,15,16,17,18,19,20,21,22,23,24,25,26,27,28,29,30,31,32,33,34,35,36,37,38,39,40,41,42]). The selected articles inserted in Table 1 deal with (i) cellulases and other important carbohydrate-active enzymes; (ii) lipases, to manipulate feedstock oils for biodiesel production and (iii) other biocatalysts, including those commercially available. However, to depict the current state of interest when only marine feedstock exploitation is present, chemical treatments were also listed in these selected modern articles (entries 13–34, Table 1).

Non-conventional sources of cellulase enzymes have been sought for a long time. As mentioned in an old report, the identification of these biocatalysts in the marine fungus *Dendryphiella arenaria* [43] dates back 40 years. However, of recent interest are the complex biopolymers in microalgae cells subjected to breakdown in biological pretreatments, as reported in a more modern review [44]. The focus is not only on cellulases as the most-explored specific and efficient enzymes (entries 1–5 Table 1), but also on other hydrolytic enzymes, including hemicellulase, pectinase, protease and amylase, even in the form of an enzymatic cocktail seen as an effective tool with respect to single enzymes as well. Entire microbial communities associated with marine organisms are studied (entry 2 Table 1). Chitinases are very well represented in research, too, and new insights into the disruption of crystalline polysaccharides were gained, as seen in a recent review report [45]. Substrate-disrupting accessory non-hydrolytic proteins are novel tools to improve molecular accessibility to polymers with increased process efficiency. Other primary articles, dealing with different carbohydrate active hydrolases, are listed in Table 1 (entries 6–8).

Obviously lipases are also considered a convenient tool for converting a wide range of feedstock oils into biodiesel [46]. A study of 427 yeast strains from seawater, sediment, mud of salterns, guts of the marine fish and marine algae should also be mentioned here [47]. Industrial yeast *Yarrowia lipolytica* of marine origin is a biocatalyst of interest in metabolic engineering studies used to expand the substrate range [48]. Entries 9–12 in Table 1 are interesting primary articles along this line of research. Microalgae cultivation and macroalgae developed as pests due to eutrophication are generally seen as potential resources for biofuel production [49]. Another interesting aspect is the combination of macroalgae cultivation exploiting nutrients coming from marine aquaculture or other processes.

In industrial squid manufacturing for chitin production, a large volume of protein effluents containing peptones from alkaline and enzymatic hydrolysis of the pens are substrates used as a nitrogen source to reduce the cost of marine probiotic bacteria cultivation (entry 13, Table 1). A similar approach was also reported for effluents originating from the industrial thermal treatment of mussels (entry 14, Table 1); many other examples on these lines are reported here (entries 15–33, Table 1), where studies on different biomasses were conducted often by comparing chemical and enzymatic procedures and yeast fermentations adopted for bioethanol production. An enzymatic cocktail of glucanase, cellulase and glucosidase was studied for R-phycoerythrin extraction, assisted by ultrasound technology from the red seaweed *G. turuturu* (entry 34, Table 1) proliferating along the French coast. 

Marine biomass-centered studies are also listed in the section about food industry development. Additionally, a particular and interesting aspect studied is the use of algicidal microorganisms to improve cell disruption during biotechnological processes aimed at producing biofuels. Secreted algicidal substances to be used as microalgae breakdown agents are reviewed [50].

## 4. Food Applications

Enzymatic procedures in food processing are mostly based on biocatalysts of terrestrial or microbial origin, with new enzymes currently obtained from these environments. For years the marine domain has been seen as a promising source of interesting biocatalysts [51] for modern applications. However, the enzymatic activities present in seafood or in byproducts were utilized for centuries in the traditional production of various cured and fermented seafood, allowing the preparation of numerous sauces and pastes from the time of the Greeks and Romans and also seafood processes in the Far East [52]. 

The recovery and processing of waste is generally a challenge in the food industry. Especially in the seafood sector, a more complete utilization of the raw material with minimization of the inherent problems of pollution and waste treatment is a current issue. Many reviews or chapters in books depicting the state of the art of this specific topic were found. One of the oldest reports found during our search dates back to 1978; interestingly, it already pointed out bioconversion tools for the processing and valorization of food waste in the conversion to useful products [53]. Upgrading of sea byproducts is, however, still central in more modern analysis [54], with more attention dedicated nowadays to therapeutic potential. Attention to antihypertensive and immunomodulatory agents (i.e., peptides obtained by enzymatic hydrolysis of fish proteins) is recognized more than the simple nutritional and biological properties of these materials. Both have recently been investigated in marine mussels [55]. While for mussel proteins the focus is on peptides obtained by bioprocesses, lipids (PUFAs) are also investigated for the prevention and treatment of rheumatoid arthritis. The use of all-natural stabilizers for food, in the form of (enzymatically) muscle-derived extracts, appears interesting, as well as the addition of plant extracts or pure phenolic compounds to combat oxidation in seafood [56]. Basic research related to the enzymatic processes occurring in seafood material has also been traced; a study of blackening processes in freeze-thawed prawns during storage is of interest. The respiratory pigment hemocyanin is converted into a phenoloxidase-like enzyme and acts as a potent inducer of post-harvest blackening; these discoveries are helpful for the development of anti-blackening treatments for these foods [57]. Biotechnologists are also interested in the large availability of seafood raw materials; byproducts from waste in processing (liver, skin, head, viscera, trimmings, etc.) amount to 60% or more [58] of total renewable raw material. Review articles were found for general aspects [59,60] and a particular focused on seaweeds [61].

The food and biorefinery sections overlap because byproducts, primarily used as feed with low returns, are also thought to be useful for biodiesel generation. Some articles could thus be listed in both sections; however, repetition is avoided here and also in this case tabulation is based on the same fundamental key for the analysis of literature adopted previously in Table 1; enzyme (marine or commercial)-based bioprocesses and types of marine-originating feedstocks are the two columns of Table 2, including a third one for notes [62,63,64,65,66,67,68,69,70,71,72,73,74,75,76,77,78,79,80,81,82,83,84,85,86,87,88,89,90,91,92,93,94,95,96]. Commercial or marine-originating proteases and lipases are most used for production in this field and a wide range of edible biomass for the valorization study is listed in Table 2. They are also an excellent, low-cost source for enzyme production [97].

An enzymatic approach can overcome the environmental impact of traditional processes and make the processes sustainable and cost-effective. In a very recent comprehensive review, it is reported that significant developments can be expected for enzyme applications in the fish and seafood industries [98] in the near future. 

## 5. Fine Chemistry and Lab Techniques

Biocatalytic procedures using marine enzymes for the production of fine chemicals are an important aspect of this review. The production and manipulation of complex biomolecules benefited from important biotechnological features characterizing biocatalyzed reactions with respect to the use of purely chemical-based methods [99].

Technological improvements (metagenomics) applied to bioprospecting in understudied environments help to identify a greater repertoire of novel biocatalysts with complementarity about properties (stereochemistry, resistance, etc.). Marine enzymes offer hyperthermostability, salt tolerance, barophilicity, cold adaptability, chemoselectivity, regioselectivity and stereoselectivity [4], thus acting as useful and new alternatives to terrestrial biocatalysts in use. Particular importance is represented by enzymes showing resistance to organic solvents, with the examples from marine environments mostly related to halophilic proteins (salt reduces water activity, like organic solvent systems), as analyzed in a recent, comprehensive review article [100]. 

As for carbohydrate-active enzymes, selective and easy manipulation of structurally complex marine polysaccharides provides homogeneous core blocks (oligosaccharides) for analysis and hemisynthesis. This constitutes the core of a sustainable process when using renewable resources. Other preminent examples of enzymatic treatments as a tool to improve the extraction efficiency of specific bioactive compounds from seaweeds were recently reviewed [101] and laboratory techniques in the preparation of compounds for further research are discussed in a recent report [102] listing enzymes for the functionalization of chitosan such as polyphenoloxidases (PPO) (tyrosinases, laccases) and peroxidases (POD); examples from the marine environment are indicated. 

Due to our specific design of search terms in querying literature databases, the coverage of articles dealing with the simple prospecting, isolation and identification of new marine biocatalyst(s) is partial; however, however those found are included in this section. Indeed, most articles listed in Table 3 [103,104,105,106,107,108,109,110,111,112,113,114,115,116,117,118,119,120,121,122,123,124,125,126,127,128,129,130,131,132,133,134] focused on bioprocesses for the synthesis of useful products and on enzymatic routes adopted for setting up laboratory techniques to study marine complex biomolecules (e.g., improving extraction, digestion of polysaccharides to simple components for structural determination, etc.). Various examples are found in the literature of the synthesis and hydrolysis of glycosidic bonds. Entry 8, Table 3 [110] is only one of the examples of synthetic strategies for the production of interesting products (enzymatically glycosylated natural lipophilic antioxidants). Polymers with synthetic carbohydrates have a wide range of applications in medical biotechnology as new biomaterials [135] and carbohydrate-active hydrolases can also be applied; moreover, these biocatalysts are important for the synthesis of a number of novel dietary carbohydrates in food technology, for the production of chromophoric oligosaccharides of strictly defined structure as valuable biochemical tools, etc. Other numerous applications oriented to vegetal waste treatment in recovering useful materials are reported in the section devoted to biorefinery. 

Tabulation of primary articles in this section (Table 3) is based on biocatalyst(s) used, product(s) obtained with the biocatalyzed process, or evaluating of marine feedstock(s); comments about the contents of the article are also reported in the notes in Table 3. Entries 1–18 (Table 3) are related to carbohydrate-active hydrolases, while a few (entries 19–23) are listed for ester hydrolysis and proteolytic activities (entries 31 and 32). 

Little is known about the distribution and diversity of *Candida* genus in marine environments, with only a few species isolated from these environments [136]. *C. rugosa* and *C. antarctica* are among the terrestrial yeasts, so biocatalysis-related articles dealing with these quite famous commercial lipases are not listed here. Interesting examples of oxidoreductases are also found in the literature, both for direct biocatalytic applications (including examples of immobilized enzymes) and for biogenetically related studies (see entries 24–30, Table 3).

Finally, it is worth mentioning in this section a remarkable review focused on the biosynthesis of oxylipins in non-mammals. Biocatalysts involved in pathways related to these biomolecules carry interesting and unusual catalytic properties for biocatalysis. A detailed biocatalytic knowledge of enzymatic catalysis in these reactions is needed to plan a possible direct in vitro biocatalytic lab-scale production of useful products [137]. With the similar aim of increasing knowledge about natural enzymes with interesting features, a review was compiled on the enzymatic breakage of dimethylsulfoniopropionate [138]. The compound, a zwitterionic osmolyte produced by corals, marine algae and some plants in massive amounts (ca. 10^9^ tons per year), is transformed by marine microbes and bioprocesses involving this molecule and derivatives are of interest for assessing the ability of relevant enzymes to realize these transformations.

## 6. Sediments and Bioremediation

Among the studies on marine sources for enzymes, the fields of marine biomarkers and bioremediation applications are of high significance in this context [139]. 

In the list of parameters that are usually considered when assessing exposure to environmental pollutants in aquatic ecosystems, biotransformation enzymes (phase I and II), biotransformation products and stress proteins are of high interest for enzymatic processing. It is of value, for example, that a unique set of protein expression (signature) for exposure to different chemical compounds has been recognized for *Mytilus edulis* and the expressed proteins identified participate in α- and β-oxidation pathways, xenobiotic and amino acid metabolism, cell signalling and oxyradical metabolism [140]. Bioremediation as an enabling technology exploits naturally occurring organisms that with their metabolic ability are able to transform toxic substances in less hazardous compounds that in turn are included in biogeochemical cycles. Stereochemical aspects play an important role due to the fact that homochirality appears to be a requirement for the functioning of enzymes with specific (partial) incorporation of stereoforms. Enantioselective chromatographic separation of chiral environmental xenobiotics is covered in an interesting review. The study includes microbial transformation of chiral pollutants in aquatic ecosystems, enzymatic transformations, etc. [141]. An additional report states the effectiveness of enzymatic processes in bioremediation even though limited application is evidenced with respect to stability and the cost of biocatalysts. Marine enzymes are seen as a solution to this challenge. In particular, marine fungi and their laccases are used in the textile industry, mostly to deal with salty effluents [142]. 

A recent report of a European project in this field characterizes novel hydrocarbon degrading microbes isolated from the southern side of the Mediterranean Sea. Exploiting and managing the diversity and ecology of microorganisms thriving in these polluted sites is a major objective in terms of increasing knowledge of the bioremediation potential of these poorly investigated sites [143].

In Table 4 primary articles in the field are selected [144,145,146,147,148,149,150,151,152,153,154,155,156,157,158,159,160,161,162,163,164,165,166,167,168], indicating the biocatalyst(s) or organism(s) exploited and details of application, with notes about the results and importance of the work.

## 7. Others

Searching within records used in this review for the names of class of enzymes, oxidoreductases, transferases, hydrolases and lyases are equally represented, while isomerases and ligases are less often used as keywords. However, only 30% of the total records contain such names for classes, with common names of biocatalysts more often adopted in titles, abstracts and keywords. Thus, in this section a few other topics, difficult to insert in previous sections, are covered.

Enzymes acting on 1,4 glycosidic bonds between galacturonic acid residues in pectin were investigated for the improvement of banana fiber processing. At least one actinomycete strain of *Streptomyces lydicus* collected from estuarine and marine areas in India was found to be a potent producer of polygalacturonase [169]. 

In a study to reduce the cost of aquaculture of fish cobia by adding crustacean processing waste, an investigation of the endogenous chitinolytic enzymes in cobia was conducted. It suggested substantial endogenous production of enzymes of the chitinolytic system and that the activity from chitinolytic bacteria was not significant [170]. 

An interesting study based on enzymology tools was conducted for demonstrating the enhanced mixing processes between the sediment and the overlying waters of the Delaware Estuary. The authors used fluorescently labeled polysaccharides to determine the effects of suspended sediment transport on water column hydrolytic activities [171]. 

In another report, a rapid and easy-to-use set, composed of semi-quantitative kits, was adopted for the investigation of the heterotrophic bacterial community in meadows of *Posidonia oceanica* during environmental surveys. Although the set is composed of known kits (ApiZym galleries, Biolog microplates and BART™ tests) principal enzymatic activities, metabolic capabilities and benthic mineralisation processes were all studied [172]. 

A particular aspect is related to carbonic anhydrases; it is worth mentioning in this review since it was recently discussed in a book devoted to these enzymes from extremophiles and the possible biotechnological applications for which they can be used [173]. Among the CO_2_ sequestration methods proposed in order to capture and concentrate CO_2_ from combustion gases, the biomimetic approach [174] could indeed benefit from the diversity of marine carbonic anhydrases. However, these metallo-enzymes are also discussed for their potential as novel biomarkers in environmental monitoring and the development of biosensors for metals [175], and another interesting aspect, encompassing at least two of the fields listed in this review, is the investigation of other enzymatic activities possessed by carbonic anhydrases [176].

## 8. Conclusions

One of the recent transnational calls of ERA-NET (ERA-MBT), an action funded under the EU FP7 program, was focused on biorefinery and entitled “The development of biorefinery processes for marine biomaterials”. The projects were required to develop the production of a large number of different products and novel processes through the application of biotechnological knowledge. Further technological developments to improve the integration and optimisation of the processing steps were required. In this realm seaweed biomass seems to have great potential as a raw material in a biobased economy, offering advantages such as no competion with food production, absence of fertilisers or pesticides in the value-chain and positive relapses removing an excess of nutrients from marine environments. However, a few key areas where development is still needed are technology for the improvement of large-scale cultivation and fractionation, and the identification of new marine microbial strains to break down macroalgal polysaccharide.

The use of macroalgae in food preparation is a classical topic in food applications found when inspecting the primary articles listed in Table 2. Macroalgae contain a high concentration of minerals, vitamins, trace elements and fibre and have low fat content. Different projects studying these aspects are in development. It is of interest that a close inspection of the affiliations of the corresponding authors of all articles in Table 2 reveals that Spain and France are the top two countries where research efforts were published; however, many countries produce interesting research results.

As for fine chemistry and laboratory techniques, different case studies recently illustrated the importance of biocatalysis, considering the specificities of marine enzymes with respect to their terrestrial counterparts [177]. Ketone reduction and epoxide hydrolases useful in organic synthesis appeared to be central for stereochemical aspects. Access to bioactive aldehydes with lipooxygenases and lipases actions and biodegradation of marine pollutants are covered, along with other lipolytic activities used for enantioselective hydrolysis. From the overall analysis and examples reported, the strategy regarding the potential of marine habitats is clear. It is important to report the note of two editors in a special issue dedicated to biocatalysis in *Current Opinion in Chemical Biology*; they stated that “At least a third of this planet’s biomass resides in the oceans, and the rules of the marine biochemical game seem to be fundamentally different than those described in our biochemistry textbooks.” [178].

In the bioremediation field, [141] is very stimulating, pointing out the importance of stereoselectivity in aquatic ecosystem biotransformations and reporting on the great impact that the use of cyclodextrins in chiral gas-chromatography had in widening this knowledge. All the information reported by the studies on processes is of great interest to biocatalysis practitioners, starting from pioneering investigations into the distribution of atropisomeric PCBs in the marine environment. Marine bacteria specialising in the degradation of hydrocarbons have been isolated from polluted seawater and some of these bacteria can grow on these substrates; they represent an extraordinary archive of mono- and dioxygenases, oxidases, dehydrogenases and other enzymatic activities that can be applied in regio- and stereoselective biocatalysis. There is a gap between the general knowledge from the studies in this section and the specificity/suitability required for preparative enzymatic processes; bridging this gap could shed more light on the useful features of the enzymes involved in this type of pollutant biotransformation, thus enabling more effective application.

Fewer than half of the results from our literature search are cited in the references list. About 20% of the hits are represented by books or reference works that were hardly used here. Additionally, only four scientific journals hosted more than 10 articles of the remaining corpus: *Bioresource Technology*, *Food Chemistry*, Process *Biochemistry* and *Chemosphere* together account fornot far off 100 articles. The top specialist marine-oriented journal was *Algal Research*, with only eight papers. Inserting representative primary articles in the tables and excluding the ones that do not belong resulted in the current total number of references. Therefore, scientific interest in marine enzymatic processing can be considered successfully published in non-specialized journals such as the ones cited above; the separation of fields, as adopted here, is only for ease of discussion. This has already been mentioned above for marine biomass-centered studies that could have been listed under food applications or biorefinery, or for enzymatic activities used for polysaccharide manipulation that could have been listed under fine chemistry and lab techniques instead of biorefinery. 

In conclusion, all these aspects point to a final consideration of the importance of an interdisciplinary network in setting up successful research projects enabling the identification of an arsenal of enzymes and pathways greatly in demand for biotechnological applications. In continuing this research effort, further refining of the scientific literature could be of interest; exploration of the fields individuated above should be continued in depth, in specialized journals, in a manner that could help to reveal sub-fields along with more details pointing to a single process with room to discuss a single enzymatic activity.

## Figures and Tables

**Table 1:** Biorefinery.

Entry	Reference	Biocatalyst(s)	Biomass	Notes
Cellulases and other important carbohydrate active hydrolases				
1/2015	[9]	Cellulase of marine fungus *Cladosporium sphaerospermum*	Seaweed biomass *Ulva fasciata*	Cellulases found are active and stable in different ionic liquids
2/2014	[10]	Lignocellulose depolymerizing multi-enzyme complex: lignin peroxidase, xylanase and cellulases		13 microbial marine isolates from seaweed were studied. They belong to the genera *Brachybacterium*, *Brevibacterium*, *Halomonas*, *Kokuria*, *Micrococcus*, *Nocardiopsis*, *Pseudomonas* and *Streptomyces*
3/2014	[11]	Cellulase from a marine bacterium, *Bacillus carboniphilus*		Optimization study of saccharification using marine microbial cellulase
4/2013	[12]	Cellulase from a marine *Bacillus* sp. H1666	*Ulva lactuca* macro algae is studied for cellulase treatment	Isolated enzyme has saccharification applicability on *Ulva lactuca*
5/2009	[13]	Cellulase isolated from a marine bacterium, *Bacillus subtilis* subsp. *subtilis* A-53		
6/2014	[14]	κ-carrageenase CgkA and λ-carrageenase CglA from *Pseudoalteromonas carrageenovora*	Carrageenan from red algae	Improvement of the process of degradation by the study of functional carrageenolytic complex
7/2016	[15]	Endo-type β-agarase AgaG1, screened from *Alteromonas* sp. GNUM1; exo-type β-agarase DagB from *S. coelicolor* A3 and a α-neoagarobiose hydrolase from *Alcanivorax* sp.	Agarose	Enzymatic agarose hydrolysis process without acid pretreatment
8/2012	[16]	Mimicked the natural agarolytic pathway using three microbial agarases (Aga16B, Aga50D and DagA) and NABH	Recalcitrant agar polysaccharide	
Feedstock oils to biodiesel				
9/2016	[17]	-	Marine microalgae	Optimization study for disruption of thraustochytrid cell using bead mill for maximising lipid extraction yields and hydrolysis of oil extracted studied using commercial lipases
10/2013	[18]	Bacterial isolate *Flammeovirga yaeyamensis*	Oil-rich microalga (*Chlorella vulgaris* ESP-1)	Cell-wall destruction analyzed by SEM micrographs was associated with the activity of hydrolytic enzymes
11/2015	[19]	*Chlorella protothecoides*		Study of fermentations developed to produce lipid by heterotrophic *C. protothecoides* using cassava bagasse as the low-cost feedstock
12/2014	[20]		*Nannochloropsis oceanica*	Crude algal oils were extracted from the oleaginous microalga *Nannochloropsis oceanica*
Marine feedstock valorization				
13/2015	[21]	Three enzymes used: alcalase, neutrase and esperase	Effluents obtained from chemical and enzymatic chitin production of *Illex argentinus* pen byproducts	Study for production of lactic acid bacteria, marine probiotic bacteria and two common gram (+) bacteria using effluents as substrates
14/2015	[22]	Chemical treatment	Mussel processing wastewaters	Laboratory optimization to transform mussel processing wastewater into a growth culture medium to produce microbial biomass. The lab-scale processes studied were upscaled to a pre-industrial level using a 70-L fermenter
15/2015	[23]	Sulfuric acid was seen as the best catalyst with a lipid conversion efficiency of 44.9%	Marine microalga *Chlorella* sp. BDUG 91771	-
16/2016	[24]	Pretreatment with hydrogen peroxide	Seaweed *Ulva prolifera*	Optimization study
17/1994	[25]	Commercial bacterial inoculum (Stabisil)	Crustacean shell waste from the world’s fishing industry	Optimization study for recovery of protein, pigment and chitin from waste shell of prawn
18/2017	[26]	Cellulase and pectinase	*Porphyridium cruentum*, red microalagae	Evaluation of bioethanol production in response culture conditions of to Porphyridium cruemtum. Enzymatic hydrolysis resulted in high glucose conversion yields for both seawater and freshwater conditions
19/2016	[27]	Commercial cellulases and alginases	Brown algae *Macrocystis pyrifera*	Study of various pretreatments
20/2016	[28]	Mixture of commercial enzymes: Viscozyme^®^ L, Cellic^®^ CTec2, Cellic^®^ HTec2	Macro-algae *Gracilaria verrucosa*	
21/2016	[29]	Chemical and enzymatic process with commercial cellulase and β-glucosidase	Red macroalgae *Eucheuma cottonii*	
22/2015	[30]	Celluclast and commercial alginate lyase (EC4.2.2.3) from *Sphingobacterium spiritivorum* were used	Brown seaweed *Laminaria digitata*	
23/2015	[31]	Commercial cellulase, xylanase and β-glucosidase	*Nannochloropsis salina*	Anaerobic digestion study
24/2015	[32]	Commercially available enzymes (pectinase) and enzyme mixtures (Accellerase 1500, Accellerase XC, and Accellerase XY) with multiple enzyme activities (exoglucanase, endoglucanase, hemi-cellulase, and β-glucosidase were used)	*Nannochloropsis salina*	Study of conversion of lipid-extracted biomass into fermentable sugars
25/2014	[33]	Commercial cellulase	Red algae *Kappaphycus alvarezii*	Optimization study
26/2014	[34]	Commercial Viscozyme L and Cellic CTec2	Marine green macro-algae *Enteromorpha intestinalis*	Study of hydrotermal method
27/2014	[35]	Free and immobilized yeast	Red alga *Gracilaria* sp.	A study for bioethanol production using hydrolisate of *Gracilaria*
28/2014	[36]	Yeast fermentation	Microalga *Chlorella vulgaris*	Pectinase enzyme was used for disrupting microalgal cells
29/2013	[37]	*Saccharomyces* fermentation	Red alga *Gracilaria verrucosa*	A study for the combined production of agar and bioethanol; the pulp was used after agar extraction
30/2013	[38]	*Saccharomyces* fermentation	Red algae *Kappaphycus alvarezii*	105 L of ethanol per ton of seaweed were obtained after a dilute acid pretreatment
31/2012	[39]	Yeast fermentation	Aquatic plant *Zostera marina*	Study of the potential of this plant as a source of bioactives and sugars for bioethanol production
32/2011	[40]	*Saccharomyces* fermentation	Byproduct from the alginate extraction process	Interesting study for exploitation of seaweed waste from alginate production
33/2015	[41]	-	Red alga *Gracilaria verrucosa*	Optimization study of this suitable feedstock for biosugar production
34/2015	[42]	β-1,3-glucanase, cellulase and β-glucosidase were studied	Red seaweed *Grateloupia turuturu*	Enzyme-assisted extraction of R-phycoerythrin with ultrasound technology

**Table 2:** Food applications.

Entry	Reference	Biocatalyst(s)	Biomass	Notes
1/2007	[62]	Antibacterial alkaline protease	Fish processing waste	Action exerted by cell lysis of pathogenic bacteria
2/2016	[63]	Alcalase for oil extraction	*Thunnus albacares* byproducts (heads)	Study for deodorization of fish oil
3/2016	[64]	Commercial alcalase	Shrimp waste	Response surface methodology study to grow hydrocarbon-degrading bacteria *Bacillus subtilis*
4/2016	[65]	Enzymatic deproteinization by commercial enzyme savinase	Norway lobster (*Nephrops norvegicus*) processing byproducts	Chitin and chitosan production
5/2016	[66]	Commercial enzymes used for the preparation of seaweed: Celluclast and Alcalase	Brown seaweed *Ecklonia radiata*	Study of extraction methods of the alga and potential in vitro prebiotic effect
6/2016	[67]	Commercial alcalase	Wastewater generated during shrimp cooking	Study for the production of enzymic hydrolysates with antioxidant capacity and production of essential amino acids
7/2016	[68]	Commercial alcalase	Head byproducts of *Prionace glauca*	Production of chondroitin sulphate from blue shark waste was studied after cartilage hydrolysis with alcalase
8/2016	[69]	Alcalase	Adhesive gum layer surrounding naturally fertilised ballan wrasse (*Labrus bergylta*) eggs	A study for the biological control (by cleaner fish *Labrus bergylta*) of sea lice in farming *Salmo salar*
9/2016	[70]	Commercial lipase B from *C. antarctica* (Lipozyme 435, immobilized lipase)	Sardine oil	Sardine oil was evaluated by glycerolysis using commercial lipase to produce monoacyl glycerols rich in omega-3 polyunsaturated fatty acids
10/2015	[71]	Proteases from *Bacillus subtilis* A26 (TRMH-A26), *Raja clavata* crude alkaline protease extract, alcalase and neutrase	Thornback ray (*Raja clavata*) muscle	Study of bioactivity of extracts after proteolytic hydrolysis with different enzyme preparations
11/2015	[72]	-	Brines marinated herring (*Clupea harengus*)	Brines from marinated herring processing used for recovery of useful material
12/2015	[73]	Commercial proteases	Atlantic salmon (*Salmo salar*) rest raw materials	Study of the production of different hydrolysates using commercial enzymes for the valorization of viscera-containing raw material from Atlantic salmon
13/2015	[74]	Marine proteases	Red scorpionfish (*Scorpaena scrofa*) viscera	Alkaline proteases of marine origin suggested for detergent formulations and deproteinization of shrimp shells
14/2015	[75]	Commercial alcalase, pepsin and trypsin	Common carp (*Cyprinus carpio*) egg	Hydrolysates improve the immune system with differential influences on the immune function. Interesting study for several applications in the health food, pharmaceutical, and nutraceutical industries
15/2014	[76]	Hydrolysis by bromelain	Protein byproducts of seaweed (*Gracilaria* sp.)	Set up of a flavouring agent with umami taste and seaweed odour
16/2014	[77]	Different commercial proteases	Fresh herring byproducts	Enzymatic hydrolysis to produce fish protein hydrolysates and separate oil
17/2014	[78]	Hydrolysis by commercial proteases	Cod (*Gadus morhua*) fillets	Study of influences of oxidative processes during protein hydrolysis using cod
18/2013	[79]	Proteolytic processing with commercial proteases	Fractions obtained from processing of Atlantic rock crab (*Cancer irroratus*) byproducts	Small peptides with biological activity recovered
19/2013	[80]	Commercial alcalase	Protein concentrates recovered from cuttlefish processing wastewater	Selective ultrafiltration methods under study for concentrating active components with antihypertensive and antioxidant activities
20/2013	[81]	Commercial alcalase	Tuna dark muscle	Basic study for fractionation of protein hydrolysates with ultrafiltration and nanofiltration
21/2012	[82]	Proteases and lipases from marine waste	Byproducts of Monterey sardine (*Sardinops sagax caerulea*) processing	Actions of enzymes from sardine byproduct (viscera and byproduct concentrate extracts) produced 3-fold greater hydrolysis than with the commercial enzyme
22/2012	[83]	Trypsin and alcalase	Waste byproducts of red seaweed *Porphyra columbina*	Study on protein water extracts wasted during traditional phycollloids extraction procedure from *P. columbina.* Interesting immunosuppressive effects and antihypertensive and antioxidant activities found
23/2012	[84]	Commercial alcalase	Fish byproducts	Comparison of methods including enzymatic extraction
24/2009	[85]	Proteolytic commercial enzyme mix	Snow crab (*Chionoecetes opilio*) byproduct fractions	Pilot scale enzymatic hydrolysis to entire snow crab byproducts followed by fractionation operations in order to recover enriched fractions of proteins, lipids and chitin
25/2009	[86]	Commercial alcalase preparations	*Gadus morrhua* skin collagen	Optimization of parameters for the hydrolysis
26/2008	[87]	Three types of enzymes used: papain, trypsin and pepsin	Wastewater from the industrial processing of octopus	Marine peptones as promising alternatives to expensive commercial medium for growth of lactic acid bacteria
27/2008	[88]	Commerical proteases	*Dosidicus gigas* mantle	Tenderization of mantle for commercial use as substitute of *Illex argentinus*
28/2007	[89]	Alcalase	Shrimp processing discards	Isolation and characterisation of a natural antioxidant from shrimp waste
29/2005	[90]	Alcalase, Lecitase^®^ a carboxylic ester hydrolase with inherent activity towards both phospholipid and triacylglycerol structures	Cod (*Gadus morhua*) byproducts	Study for protein and oil extractions
30/2005	[91]	Flavourzyme, a fungal protease/peptidase complex produced by *Aspergillus oryzae*, and Neutrase	Cod (*Gadus morhua*) byproducts	Composition of products generated by hydrolyses of byproducts of cod processing for optimization and design on desired product
31/2003	[92]	Crude papain was selected to perform the enzymatic extraction	Skate cartilage	Study for a low-cost process for glycosaminoglycan extraction from skate cartilage
32/2002	[93]	Umamizyme (commercial endo-peptidase activity from a strain of *A. oryzae*)	Tuna waste	Study for evaluation of activity of Umamizyme in comparison to other fungal enzymes
33/2001	[94]	Alcalase	Yellowfin tuna (*Thunnus albacares*) waste	Study of hydrolysis of tuna stomach proteins
34/2016	[95]	Six commercial enzyme mixtures and individual enzymes were used: Viscozyme^®^ L, Celluclast^®^ 1.5 L, Ultraflo^®^ L and the three proteases Alacalase^®^ 2.4 L FG, Neutrase^®^ 0.8 L and Flavourzyme^®^ 1000 L.	Brown alga *Ecklonia radiata*	Study of enzyme-assisted extraction of carbohydrates for the design and optimization of processes to obtain oligo- and polysaccharides
35/2014	[96]	Proteolytic preparations from *Bacillus mojavensis* A21, *Bacillus subtilis* A26, *Bacillus licheniformis* NH1, *B. licheniformis* MP1, *Vibrio metschnikovii* J, *Aspergillus clavatus* ES1 and crude alkaline protease extracts from Sardinelle (*Sardinella aurita*), Goby (*Zosterisessor ophiocephalus*) and Grey triggerfish (*Balistes capriscus*) prepared and characterized by the group	Shrimp processing byproducts	Enzymatic deproteinization for extraction of chitin

**Table 3:** Fine chemistry and laboratory techniques.

Entry	Reference	Biocatalyst(s)	Product(s)	Feedstocks	Notes
Carbohydrate active hydrolases					
1/2016	[103]	Alkaline β-agarase from marine bacterium *Stenotrophomonas* sp. NTa	From agarose as substrate neoagarobiose, neoagarotetraose and neoagarohexaose are the predominant products	-	First evidence of extracellular agarolytic activity in *Stenotrophomonas*, the enzyme exhibited stability across a wide pH range and resistance against some inhibitors, detergents and denaturants
2/2016	[104]	Cloned novel chitinase from a marine bacterium *Paenicibacillus barengoltzii* functionally expressed in *E. coli*	The chitinase hydrolyzed colloidal chitin to yield mainly *N*-acetyl chitobiose	Chitin (from crab shells)	Production of 21.6 mg·mL^−1^ of *N*-acetyl chitobiose from colloidal chitin with the highest conversion yield of 89.5% (*w*/*w*)
3/2015	[105]	Chitinase from the marine-derived *Pseudoalteromonas tunicata* CCUG 44952T	Active also on chromogenic substrate pNP-(GlcNAc) but not on pNP-(GlcNAc)_2_ and pNP-(GlcNAc)_3_	Colloidal and crystalline chitin	The recombinant enzyme exhibited antifungal activity against phytopathogenic and human pathogenic fungi, (biofungicide)
4/2014	[106]	Commercial pectinase or acidic hydrolysis	3-deoxy-d-*manno*-oct-2-ulosonic acid (Kdo): a sugar that is difficult to obtain by chemical synthesis and that has applications in medicinal chemistry	Marine microalgae, *Tetraselmis suecica*	Evaluation of *T. suecica* as feedstock for a KDO production
5/2014	[107]	α-amylase from marine *Nocardiopsis* sp. strain B2	-	-	Study for immobilization of a marine α-amylase by ionotropic gelation technique using gellan gum (GG)
6/2014	[108]	Endo- and exo-glucanases from marine sources: endo-1,3-β-d-glucanase (LIV) from *Pseudocardium sacchalinensis* and the exo-1,3-β-d-glucanase from *Chaetomium indicum*	Different fractions of oligosaccharides	Laminaran from brown alga *Eisenia bicyclis*	Study for anticancer activity of the native laminaran and products of its enzymatic hydrolysis
7/2014	[109]	Amylolytic system in the digestive fluid of the sea hare, *Aplysia kurodai*	Maltotriose, maltose, and glucose	Sea lettuce (*Ulva pertusa*)	Enzymatic analysis of the amylolytic system in the digestive fluid of the sea hare *Aplysia kurodai* and efficient production of glucose from sea lettuce
8/2012	[110]	α-glucosidase from *Aplysia fasciata*	Glucosylated anti-oxidant derivatives of hydroxytyrosol	-	Biocatalytic production of mono- and disaccharide derivatives at final concentrations of 9.35 and 10.8 g/L of reaction
9/2006	[111]	Endo-1,3-β-d-glucanases (laminarinases) from marine mollusks *Spisula sacchalinensis* and *Chlamys albidus*	Biologically active 1,3;1,6-β-d-glucan, called translam	Hydrolysis of laminaran	Study of immobilization
10/2006	[112]	Commercial enzymes	*N*-acetyl chitobiose	Various chitin substrates α-chitin from shrimp waste	Experimental conditions studied to achive 10% *N*-acetyl chitobiose
11/2004	[113]	1→3-β-d-glucanase LIV from marine mollusk *Spisula sacchalinensis* and α-d-galactosidase from marine bacterium *Pseudoalteromonas* sp. KMM 701	Oligo- and polysaccharide derivatives possessing immunostimulating, antiviral, anticancer and/or radioprotective activity	Laminaran from the brown seaweeds *Laminaria cichorioides*	Immobilization study
12/1996	[114]	Chitin degrading enzymes from sea water bacterium strain identified as *Alteromonas*	β-(1→6)-(GlcNAc)_2_	Chitin and chito-oligosaccharides	High transglycosylation activity of the enzyme preparation was also confirmed
13/2016	[115]	Endolytic alginate lyases	4-deoxy-l-erythro-5-hexoseulose uronic acid	Alginate and alginate oligosaccharides	In depth study of degradation process from alginate to unsaturated monosaccharides
14/2015	[116]	Ulvan-degrading bacterial β-lyase from a new *Alteromonas* species	Sulfated oligosaccharides from the seaweed *Ulva*	Ulvan	Fractions of molecular weight down to a 5 kDa of oligosaccharides mix are obtained
15/2014	[117]	Extracellular β-agarases from *Agarivorans albus* OAY2	Neoagarobiose NA_2_, neoagarotetraose NA_4_ and neoagarohexaose NA_6_		Report about enzyme purification and oligosaccharides preparation
16/2007	[118]	Glycosyl hydrolases in crude extracts from extremophilic marine bacterium *Thermotoga neapolitana* (DSM 4359)	(β-1,4)-xylooligosaccharides of 1-hexanol, 9-fluorene methanol, 1,4-butanediol and geraniol	-	Transglycosylation reactions by xylose, galactose, fucose, glucose and mannose enzymatic transfers
17/2012	[119]	Commercial α-amylase	Carrageenan-derived oligosaccharide	Hydrolysis of κ-carrageenan	
18/2007	[120]	β-*N*-acetyl-d-glucosaminidase from prawn *Penaeus vannamei*			Mechanistic and inhibition studies
Ester hydrolysis					
19/1995	[121]	Fungal deacetylase	Hexa-*N*-deacetylchitohexaose	Natural or artificial chitin substrates as well as *N*-acetylchito-oligosaccharides	Enzymatic deacetylation: methodological study
20/2012	[122]	Commercial immobilized lipase, lipozyme from *Thermomyces lanuginosa*	Diglycerides and monoglycerides containing polyunsaturated fatty acids	Menhaden oil	Enzymatic ethanolysis of menhaden oil
21/2010	[123]	Organism isolated from marine sediments	Fatty acid-based biopolymer	Triglycerides of sunflower, soybean, olive, sesame and peanut as substrates	Hydrolysis of triglycerides and dimerization of fatty acid to anhydrides and subsequent formation of a Fatty acid based biopolymer (FAbBP)
22/2007	[124]	Commercial enzymes	Acylglycerol synthesis	*N*-3 PUFA from tuna oil	-
23/2016	[125]	Novel marine microbial esterase PHE14	Asymmetric synthesis of d-methyl lactate by enzymatic kinetic resolution	Racemic methyl lactate commercially available	Esterase PHE14 exhibited very good tolerance to most organic solvents, surfactants and metal ions
Oxidoreductases					
24/2014	[126]	Lipoxygenase/hydroperoxide lyase	Polyunsaturated aldehydes: 2,4,7-decatrienal and 2,4-decadienal	Macroalgal genus *Ulva* (*Ulvales*, *Chlorophyta*)	*Ulva mutabilis* is selected as cultivable for production
25/2012	[127]	Marine fungi *Aspergillus sclerotiorum* CBMAI 849 and *Penicillium citrinum* CBMAI 1186	Reduction of 1-(4-methoxyphenyl)-ethanone to its stereochemical pure alcohol (ee > 99%, yield = 95%)		Immobilization study
26/2003	[128]	Hydrogenase	Enzymatic production and regeneration of NADPH		6.2 g·L^−1^ NADPH produced with a total turnover number (ttn: mol produced NADPH/mol consumed enzyme) of 10,000
27/2003	[129]	Lipoxygenase–hydroperoxide lyase pathway	C6 and C9 unsaturated aldehydes	Brown alga *Laminaria angustata*	Study of biosynthetic pathway
28/2004	[130]	Cultures of the haptophyte microalga *Chrysotila lamellosa*	Alkanediones	Regiospecific oxygenation of alkenones	Biogenetic study
29/1996	[131]	Enzymatic extract of the marine gorgonian *Pseudopterogorgia americana*	9(11)-secosteroids	Cholesterol, stigmasterol and progesterone	Claimed as the first chemoenzymatic preparation of a natural product using the enzymatic machinery of a marine invertebrate
30/2012	[132]	Bromoperoxidase of brown alga *Ascophyllum nodosum*	4-bromopyrrole-2-carboxylate	Bromination of methyl pyrrole-2-carboxylate in bromoperoxidase II-catalyzed oxidation	Bromoperoxidase II mimics biosynthesis of methyl 4-bromopyrrole-2-carboxylate, a natural product isolated from the marine sponge *Axinella tenuidigitata*
Proteolytic activities					
31/2004	[133]	Proteolytic enzymes	Products of proteolysis	Gastric fluid of the marine crab, *Cancer pagurus*	Influence of metal ions and organic solvents other than pH and temperature are analyzed, including long-term stability over a period of several months
32/2006	[134]	Alkaline serine protease		Marine gamma-Proteobacterium	Activity in presence of up to 30% NaCl. Water miscible and immiscible organic solvents like ethylene glycol, ethanol, butanol, acetone, DMSO, xylene and perchloroethylene enhance as well as stabilize the enzyme activity

**Table 4:** Sediments and bioremediation.

Entry	Reference	Biocatalyst(s)/Organism(s)	Application(s)	Notes
1/2016	[144]	Marine microbial community for manganese oxidation	-	Several new genera associated with Mn(II) oxidation were found in metal-contaminated marine sediments and are seen as a solution for metal bioremediation
2/2013	[145]	Marine sedimentary bacterial communities	Anaerobic degradation of mixtures of isomeric pristenes and phytenes	Several bacterial products of transformation confirm the key role played by hydration in the metabolism of alkenes
3/2010	[146]	MAP kinase signaling pathway	-	Different pollutants generated different patterns of induction of the biomarker MAPK phosphorylation
4/2003	[147]	Isolate (isolate TKW) of sulfate-reducing bacteria	Reduction of chromate (CrO_4_^2−^)	Soluble hexavalent chromium (Cr^6+^) enzymatically transformed into less toxic and insoluble trivalent chromium (Cr^3+^) with potential in bioremediation of sediments contaminated by metals
5/2013	[148]	Giant freshwater prawn *Macrobrachium rosenbergii*	Study for potential biomarkers of exposure to organophosphorus pollutants: molecular and immunological responses	Investigation on the effects of the pesticide trichlorfon used in aquaculture, on molecular and enzymatic processes related to the response of the giant freshwater prawn, *Macrobrachium rosenbergii*
6/1985	[149]	Five fish species: *Salmo gairdneri*, *S. trutta*, *Galaxias maculatus*, *G. truttaceus* and *G. auratus*	Study of detoxication enzyme activities for a fungicide, chlorothalonil	Metabolism of chlorothalonil
7/1986	[150]	-	Role of enzymatic processes in the metabolism of organic matter	Proteolysis and aerobic oxidation of organic material
8/1992	[151]	-	Study for distinction between enzymatic and non-enzymatic degradation pathways in marine ecosystems	Enzymatic degradation pathways for α-hexachlorocyclohexane
9/1995	[152]	Antioxidant enzymes in mussel, *Mytilus galloprovincialis*	Use of mussels as bioindicators in monitoring heavy metal pollution	Adaptation as a compensatory mechanism in chronically polluted organisms was found
10/1995	[153]	Dioxygenase	Dioxygenase pathway with subsequent conjugation and excretion	Metabolism of benzo[a]pyrene in a freshwater green alga, *Selenastrum capricornutum*
11/2000	[154]	Polychaete worms	Sulphide detoxification by polychaete worms *Marenzelleria viridis* (Verrill 1873) and *Hediste diversicolor* (O.F. Müller)	Detoxification end-product is thiosulphate
12/2001	[155]	-	-	Biodegradation of extracellular organic carbon by bacteria in sediments
13/2004	[156]	Antioxidant enzyme activities and lipid peroxidation in the gills of the hydrothermal vent mussel *Bathymodiolus azoricus*	Study of enzymatic defences (superoxide dismutase (SOD), catalase (CAT), total glutathione peroxidase (Total GPx) and selenium-dependent glutathione peroxidase (Se–GPx) and lipid peroxidation *against metals*	Assessment of physiological adaptation to continuous metal exposure in natural environment
14/2004	[157]	Marine glycosidases	Biostimulation of enzymatic activities (glycosidases) by oxygen supply	Enzymatic activity increased when oxygenation was increased and the supply of oxygen into the sediment enhanced enzymatic degradation rates
15/2005	[158]	Antioxidant enzymes in a model organism *Daphnia magna*	Study of age-related biochemical changes in aquatic organism	General evaluation of importance of oxidative stress in aging
16/2006	[159]	European eel (*Anguilla anguilla*) exposed to persistent organic pollutants	Detection of early warning responses to pollutant exposition	Metabolic responses including detoxification mechanisms (biotransformation, antioxidant process) in European eel (*Anguilla anguilla*) exposed to persistent organic pollutants
17/2007	[160]	Catalase, superoxide dismutase, glutathione peroxidase, glutathione reductase, glutathione *S*-transferases in wild populations of mussels (*Mytilus galloprovincialis*)	Study of biochemical response to to petrochemical environmental contamination	Environmental monitoring programmes to get data that could be used as a baseline reference during oil accidents
18/2007	[161]	Acetylcholinesterase, catalase, and glutathione-S-transferase (GST) of blue mussels (*Mytilus edulis*)	Study of specific reaction to exposure to nodularin	Acetylcholinesterase activity, catalase (CAT) activity and glutathione-S-transferase (GST) in blue mussels (*Mytilus edulis*) exposed to an extract made of natural cyanobacterial mixture containing toxic cyanobacterium *Nodularia spumigena*
19/2007	[162]	Induction of biotransformation enzymes in *Sparus aurata*	Relationship between specific molecular processes (induction of enzymes) and the behavioral performance of fish is of great interest in understanding the impact of PAHs at increasing levels of biological complexity	The study investigates biochemical response to phenantrene in *Sparus aurata*
20/2012	[163]	Tow deep-sea fish species, namely *Alepocephalus rostratus* and *Lepidion lepidion* and the decapod crustacean *Aristeus antennatus*	-	Study of hepatic biomarkers (ethoxyresorufin-*O*-deethylase, EROD, pentoxyresorufin-*O*-deethylase PROD, catalase CAT, carboxylesterase CbE, glutathione-S-transferase GST, total glutathione peroxidase GPX and glutathione reductase demonstrating seasonal variation despite constant temperature and salinity
21/2013	[164]	*Platichthys flesus*	Transcriptomic study	Assessment of hepatic transcriptional differences between fish exposed to mixture of brominated diphenyl ethers and controls
22/2015	[165]	Peptidase activities	A study of enzymes using labeled substrate in diverse regions of the ocean	Enzymatic capabilities differ in Pelagic–benthic environments, affecting the processing of marine organic matter
23/2015	[166]	Arginine kinase of the crustacean *Exopalaemon carinicauda*	Bioinformatic study. Insights on the role of Cr^3+^ on enyzme with respect to inhibition and aggregation with structural disruption	An investigation of the effect of Cr^3+^ on enzymes of seawater organisms providing information on the physiological role of metal pollution in marine environments
24/2015	[167]	Oxidoreductases and catalases in *Bacillus safensis*	Isolation and enzyme identification study	The organism is responsible for degradation of the petroleum aromatic fractions
25/2016	[168]	Dehydrogenase activity or peroxidase activity	A surface methodology study	Degradation of crude oil fitted linearly with increasing biomass and enzyme activities with growth
